# Health-related quality of life of patients with rheumatoid arthritis on tocilizumab, adalimumab, and etanercept in Saudi Arabia: a single-center cross-sectional study

**DOI:** 10.3389/fphar.2023.1299630

**Published:** 2023-12-15

**Authors:** Amjad M. Alotaibi, Areej S. Albahdal, Norah Abanmy, Monira Alwhaibi, Yousif Asiri, Yazed AlRuthia

**Affiliations:** ^1^ Department of Clinical Pharmacy, College of Pharmacy, King Saud University, Riyadh, Saudi Arabia; ^2^ Pharmacoeconomics Research Unit, Department of Clinical Pharmacy, College of Pharmacy, King Saud University, Riyadh, Saudi Arabia

**Keywords:** monoclonal antibody therapy, Saudi Arabia, cross-sectional study, comparative effectiveness, health-related quality of life

## Abstract

**Purpose:** The aim of this study was to assess the quality of life (QOL) of patients with rheumatoid arthritis (RA) on tocilizumab *versus* other commonly used monoclonal antibodies (mAbs) (e.g., adalimumab and etanercept).

**Methods:** This was an interview-based cross-sectional study. Adult RA patients (≥18 years) with a disease duration of at least 1 year were included in the study. The Arabic version of EUROQOL (EQ-5D-5L) was used to assess the QOL of RA patients on mAbs. Multiple linear regression was conducted to examine the impact of tocilizumab *versus* other mAbs on the visual analog scale (VAS) score controlling for age, disease duration, treatment duration, gender, number of comorbidities, and the use of steroids.

**Results:** The number of patients who met the inclusion criteria and consented to be interviewed was 105 patients (tocilizumab (n = 37), adalimumab (n = 31), and etanercept (n = 37)). No significant differences in the scores of the five EQ-5D-5L domains across different mAbs was found. Although the VAS score of patients on tocilizumab was numerically higher compared to their counterparts on adalimumab or etanercept (69.19 vs. 62.79) this was not statistically significant controlling for age, gender, disease and treatment durations, number of comorbidities, and the utilization of steroids (*β* = 4.26, 95% CI: [-8.84–17.36], *p* = 0.52).

**Conclusion:** The use of tocilizumab did not seem to result in better QOL among RA patients. Future studies with larger sample sizes and more robust designs should be conducted to confirm or refute these findings.

## Introduction

Rheumatoid arthritis (RA) is a debilitating autoimmune disease characterized by inflammation that significantly impairs the immune system. This systemic inflammation often results in persistent pain, swelling, stiffness, and an overall reduction in functional capability, leading to psychological distress (Janke et al., 2020). Globally, RA affects an estimated 0.5%–1.1% of the population with an annual incidence rate of 0.02%–0.05% (Alamanos and Drosos, 2005). Moreover, its prevalence varies considerably across different geographic regions. For example, in Africa and the Middle East, the prevalence ranges from 0% in Nigeria to a high of 2.8% in Morocco (Almoallim et al., 2021).

In Saudi Arabia, the situation is somewhat nebulous due to the lack of accurate statistics regarding the prevalence of RA. Nonetheless, it has been observed that the incidence of the disease tends to increase with age and is nearly twice as prevalent in women as compared to men (Al-Dalaan et al., 1998).

Over the years, researchers have identified various genetic risk factors that predispose individuals to RA. The human leukocyte antigen (HLA) system is one such example, known to increase the risk of developing RA threefold, thereby making it the most prevalent and significantly associated genetic risk factor for RA (Deane et al., 2017; Petrovská et al., 2021). However, genetic susceptibility alone does not determine the onset of RA. Other non-genetic factors also come into play, including female sex, family history, lifestyle factors such as smoking, exposure to silica and textile dust, conditions like periodontitis, nutritional deficiencies, obesity, hormonal factors, lower educational level, and chronic medication use (Deane et al., 2017; Petrovská et al., 2021).

With the aforementioned, it is the intricate interplay of these environmental risk factors with an individual’s genetic background that can precipitate the breakdown of immunological tolerance, ultimately resulting in the onset of systemic autoimmunity (Deane et al., 2017; Petrovská et al., 2021). The ultimate aim of RA treatment is to achieve disease remission using treat-to-target strategy, defined as a disease activity score (DAS28) of less than 2.6, or at least low disease activity (DAS28 between 2.6 and 3.2) if remission cannot be attained (Messelink MA. et al., 2023). Furthermore, the treatment should also focus on preventing further joint damage, disability, and other systemic manifestations including cardiovascular damage (Messelink MA. et al., 2023; Messelink M. A. et al., 2023).

The current therapeutic arsenal for RA includes non-steroidal anti-inflammatory drugs (NSAIDs), glucocorticoids, as well as conventional and targeted or biological disease-modifying anti-rheumatic drugs (DMARDs) (Messelink M. A. et al., 2023). Among these, methotrexate (MTX) is often the first-line treatment due to its low cost and well-established efficacy and safety (Burmester and Pope, 2017). Conversely, biologic DMARDs—comprising of tumor necrosis factor (TNF) inhibitors, co-stimulation modifiers, interleukin-6-inhibitors, and B-cell depleting drugs—are reserved for patients who have failed to respond to or are intolerant to conventional DMARDs due to their high cost (Hsieh et al., 2020; Messelink M. A. et al., 2023). Although not enforced, the American College of Rheumatology (ACR) treatment guidelines are largely adopted by different healthcare institutions in Saudi Arabia. These guidelines recommend starting the treatment for RA with hydroxychloroquine or other conventional synthetic DMARDs, such as methotrexate or sulfasalazine for patients with low disease activity (Fraenkel et al., 2021). Moreover, the ACR guidelines favor sulfasalazine over methotrexate among patients with low disease activity, and recommends against adding a short course of glucocorticoids for patients with moderate or high disease activity (Fraenkel et al., 2021). However, the Saudi Society for Rheumatology has recently published their adapted guidelines based on the ACR treatment guidelines for RA in which they support the addition of a short course of glucocorticoids for patients with moderate or high disease activity (Omair et al., 2022). Moreover, the Saudi Society for Rheumatology recommends adding biological DMARDs if patients were unresponsive or did not reach the target after 3–6 months of treatment with conventional synthetic DMARDs, such methotrexate. Yet, the society did not recommend any specific biological DMARDs, and advise to use the least expensive one. However, it recommends using anti-TNF, such as certolizumab, as monotherapy for female patients planning to get pregnant (Omair et al., 2022).

In RA patients, disease severity and the accompanying psychological distress have a profound negative impact on their health-related quality of life (HRQoL) (Haridoss et al., 2021). As a result, RA patients often exhibit substantially lower physical, mental, and social functioning domains of HRQoL as compared to the general population (Fraenkel et al., 2021; Omair et al., 2022). Moreover, failure to manage the disease at its early stages can also adversely affect the individual’s productivity and employment prospects (Gerhold et al., 2015; Katchamart et al., 2019; Xavier et al., 2019; Haridoss et al., 2021).

TNF inhibitors, such as adalimumab (ADA) and etanercept (ETA), and other classes of biological therapies, including IL-6 inhibitors like tocilizumab (TCZ), CTLA-4 inhibitors like abatacept (ABA), and anti-CD20 agents like rituximab (RTX), have been shown to significantly improve the quality of life and symptoms in RA patients (Gerhold et al., 2015). In an observational, longitudinal, real-life study that was conducted in Bulgaria between 2012 and 2020, and compared disease activity, quality of life, and cost of different DMARDs including biological DMARDs, an improvement in both disease control and quality of life based on the clinical disease activity index (CDAI) scores and EuroQuol 5D-3L (EQ5D) was noticed. In addition, no significant difference was noticed between different biological therapies in terms of both disease control and disease activity (Tachkov et al., 2021). In addition, the utilization of biological therapies for the management of RA has shown to improve the HRQoL as measured by the EQ5D among a sample of patients in Hungary, however, when compared with conventional therapies like methotrexate, the incremental improvement in HRQoL in RA patients undergoing biological therapies was not always statistically significant (Inotai et al., 2012).

In Saudi Arabia, the use of biologic DMARDs—including ADA, TCZ, and ETA—for RA management is commonplace (Almudaiheem et al., 2018). Yet, no study to date has evaluated the impact of these biologic DMARDs on HRQoL in patients. This gap in research is particularly noteworthy considering the significant cost burden of DMARDs, accounting for almost two-thirds of the direct medical costs per a local budget impact model (Almudaiheem et al., 2018).

Moreover, the lack of concrete evidence about the incremental value of relatively more expensive biological therapies, such as TCZ, in terms of HRQoL improvement in the management of RA based on real-world data from the Middle-East underscores the need to explore biological therapies’ impact on HRQoL among RA patients in this part of the world, especially when compared with cheaper alternatives like ADA and ETA. Consequently, this study aims to compare HRQoL among RA patients administered with TCZ, against those treated with ADA or ETA.

## Material and methods

### Study design and population

This was a single-center cross-sectional study that compared the HRQoL of patients with RA on TCZ, ADA, or ETA. The study took place at King Khalid University Hospital, which is a university affiliated tertiary care center with over 900 beds. Patients aged 18 years or older with a clearly documented diagnosis of RA in their electronic health records (EMRs), and with no previous history of treatment with other biologic therapies, such as infliximab, were included. Patients who have been treated with biologic therapies other than TCZ, ADA, or ETA, such as B-cell inhibitors (e.g., rituximab or belimumab), were excluded. Moreover, patients aged <18 years, pregnant women, those with a cancer diagnosis, mental illness (e.g., depression, schizophrenia, bipolar disorders) and patients with current or previous active infection while on treatment were excluded. In addition, those treated with other DMARDs, such as methotrexate, with the exception of steroids (e.g., prednisone) were excluded.

### Data collection and study variables

The data collection has started on 23 June 2022 and ended on 17 February 2023. Data on the patient’s demographics (e.g., age, gender), weight, treatment and disease duration, baseline inflammatory markers including erythrocyte sedimentation rate (ESR), baseline C-reactive protein (CRP), medication use including TCZ or any other biological therapies, glucocorticoids, or NSAIDS, comorbidities (e.g., such as hypertension, diabetes mellitus, dyslipidemia, cardiovascular disease, asthma), and pain and morning stiffness were collected from the patients’ EMRs. Two pharmacy interns were involved in reviewing the EMRs of patients with RA. Those who met the inclusion criteria were contacted to explain to them the purpose of the research and check their willingness to participate. Those who verbally consented to participate were asked about the right date/time that suit their schedule to be contacted by telephone to assess their HRQoL using the Arabic version of the EuroQol 5-dimensions 5-levels questionnaire (EQ-5D-5L) (Harrold et al., 2018), which evaluates five dimensions of health (mobility, self-care, usual activities, pain/discomfort, and anxiety/depression) on five levels (no, slight, moderate, severe, and extreme problems), and the visual analog scale (VAS) of the EQ-5D which provides global self-assessment of health with a range between 0 (worst imaginable health) to 100 (best imaginable health). The EQ-5D-5L has been used extensively among patients with RA with lower scores being associated with severe disease activity (Harrold et al., 2018).

### Statistical analysis

The minimum sample size was estimated to be 90 patients based *α* = 0.05, *β* = 0.8, effect size d = 0.6, and power of 80%. Descriptive statistics (i.e., mean, standard deviation, frequencies, and percentages) as well as Chi-square test, Fisher’s exact test, and one-way ANOVA were used as appropriate to present and compare the sociodemographic and clinical characteristics between patients with RA on tocilizumab, adalimumab, and etanercept. Multiple linear regression was conducted to examine the impact of TCZ *versus* other biologics on the VAS score controlling for age, gender, disease and treatment durations, number of comorbidities, and the use of steroids. The statistical analysis was conducted using SAS^®^ version 9.4 (SAS institute, Cary, NC, U.S.).

### Ethical approval of the study

Only patients who verbally consented to participate were contacted and included in the study. Participants were informed about their right to withdraw from the study at any time. No personal identifiers were collected, and the collected data were stored in a safe and secure place. The study adhered to the ethical principles of the declaration of Helsinki. Additionally, the study protocol was approved by the research ethics committee of the College of Medicine at King Saud University, Riyadh, Saudi Arabia (project no. E-22-6692), and the need for written consent form was waived since the study did not collect self-reported personal identifiers or medical information and only assessed patients’ HRQoL.

## Results

**FIGURE 1:**
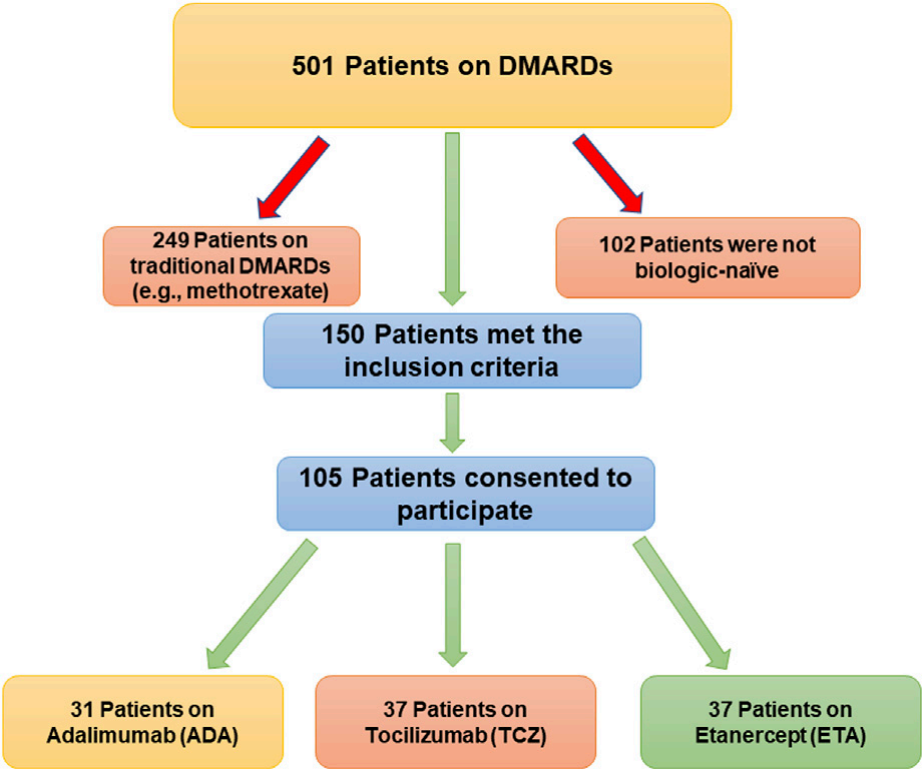
A flow diagram of patients recruitment.

**FIGURE 2:**
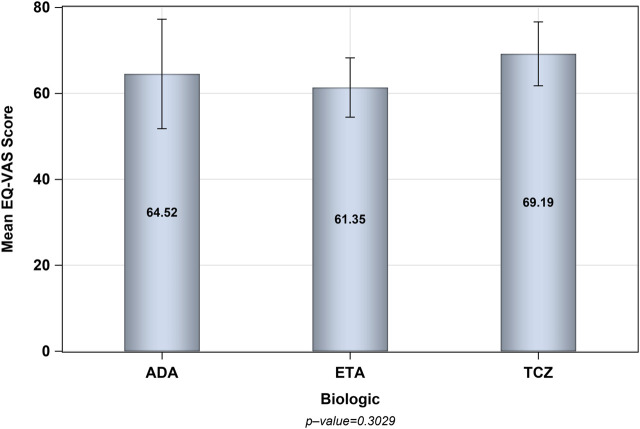
The mean scores of EQ-5D VAS for ADA, ETA, and TCZ.

The number of patients who met the inclusion criteria and were contacted was 150, however, only 105 patients consented to participate and were included in the analysis (31 on ADA, 37 on ETA, and 37 on TCZ) as shown in Figure 1. The administered dosages for ADA, ETA, and TCZ were 40 mg subcutaneous (SC) every 2 weeks, 50 mg SC once weekly, and 162 mg SC every 2 week. All of the patients were Saudi from homogeneous genetic background, and most of the participants were female (94.29%) with a mean age of 54 years and with one or no other comorbidities, such as diabetes and hypertension. However, patients on etanercept had a mean duration of illness of almost 14 years compared to 9.4 years and 10.9 years for ADA and TCZ (*p*-value = 0.0241), respectively. Additionally, patients treated with ADA or ETA had a longer mean duration of treatment compared to their counterparts on TCZ (*p*-value < 0.0001). The percentages of patients treated with steroids (e.g., prednisone 5–10 mg daily) were similar among those treated with ADA, ETA, and TCZ as shown in Table 1. The participants’ HRQoL as measured by the EQ-5D-5L which assesses five domains (mobility, self-care, usual activities, pain/discomfort, and depression/anxiety) are shown in Table 2. Although the percentage of patients with moderate to extremely severe mobility problems was 27.03% among those treated with TCZ in comparison to 35.48% and 35.13% among their counterparts on ADA and ETA, respectively, this difference was not statistically significant (*p*-value = 0.424). On the other hand, the percentage of patients with moderate to extremely severe self-care among patients on ETA was lower (5.41%) compared to their counterparts on ADA (9.68%) and TCZ (18.92%), however, this difference was not statistically significant (*p*-value = 0.296). In addition, the percentages of patients with moderate to extremely severe usual activities problems were lower among those treated with ETA (35.13%) or ADA (35.48%) compared to their counterparts on TCZ (43.25%), but this difference was not statistically significant (*p*-value = 0.389). About 35% of patients treated with ADA suffered moderate to extremely severe pain or discomfort in comparison to 54.06% and 37.84% among those treated with EDA and TCZ, respectively, but this difference was again not statistically significant (*p*-value = 0.4253). With regard to depression and/or anxiety, the percentages of patients who had moderate to extreme depression and/or anxiety were 29.04%, 10.81%, 16.22% among those treated with ADA, ETA, and TCZ, respectively, however, this difference was not statistically significant (*p*-value = 0.268). Likewise, no difference in the mean EQ-5D VAS scores for TCZ, ADA, and ETA was found (*p*-value = 0.3029) as shown in Figure 2. Controlling for age, gender, treatment duration, disease duration, number of comorbidities, and use of corticosteroids (e.g., prednisone), the use of TCZ for the management of RA did not result in higher EQ-5D VAS score, which indicates better HRQoL, compared to those treated with ADA or ETA (β-estimate = 4.26, 95% CI [–8.84–17.36], *p*-value = 0.5201) as shown in Table 3.

**TABLE 1:** Patient baseline characteristics (n = 105).

Variable	ADA (n = 31)	ETA (n = 37)	TCZ (n = 37)	*p*-value	Total
**Age**, mean ± SD	53.2 ± 15.8	54.2 ± 11.3	53.3 ± 10.6	0.9289	53.61 ± 12.43
**Gender, n (%)**					
Male	1 (3.23)	4 (10.81)	1 (2.70)	0.3687	6 (5.71)
Female	30 (97.77)	33 (89.19)	36 (97.30)		99 (94.29)
**Disease duration (yrs.)**, mean ± SD	9.4 ± 5.9	13.8 ± 6.9	10.9 ± 5.9	0.0241	11.48 ± 6.51
**Treatment duration (yrs.)**, mean ± SD	4.3 ± 2.5	5.9 ± 2.5	2.3 ± 1.3	<0.0001	4.14 ± 2.64
**Number of comorbidities, n(%)**					
0–1	24 (77.42)	27 (72.97)	29 (78.38)	0.8804	80 (76.19)
≥2	7 (22.58)	10 (27.03)	8 (21.62)		25 (23.81)
Use of steroids (e.g., prednisone)	3 (9.68)	2 (5.41)	3 (8.11)	0.8993	8 (7.62)

Bold values are indicate the *p*-values <0.05.

**TABLE 2:** Health-related quality of life assessment of ADA, ETA, and TCZ.

Items	Adalimumab (n = 31)	Etanercept (n = 37)	Tocilizumab (n = 37)	*p*-value	Total
**EQ-5D-5L**					
**Mobility**					
No problems	13 (41.94)	15 (40.54)	15 (40.54)	0.4242	43 (40.95)
Slight problems	7 (22.58)	9 (24.32)	12 (32.43)		28 (26.67)
Moderate problems	4 (12.90)	8 (21.62)	8 (21.62)		20 (19.05)
Severe problems	4 (12.90)	5 (13.51)	2 (5.41)		11 (10.48)
Extreme problems	3 (9.68)	0 (0.0)	0 (0.0)		3 (2.86)
**Self-care**					
No problems	24 (77.42)	32 (86.49)	25 (67.57)	0.2960	81 (77.14)
Slight problems	4 (12.90)	3 (8.11)	5 (13.51)		12 (11.43)
Moderate problems	1 (3.23)	0 (0.0)	3 (8.11)		4 (3.81)
Severe problems	0	2 (5.41)	3 (8.11)		5 (4.76)
Extreme problems	2 (6.45)	0 (0.0)	1 (2.70)		3 (2.86)
**Usual activities**					
No problems	12 (38.71)	12 (32.43)	12 (32.43)	0.3898	36 (34.29)
Slight problems	8 (25.81)	12 (32.43)	9 (24.32)		29 (27.62)
Moderate problems	3 (9.68)	9 (24.32)	10 (27.03)		22 (20.95)
Severe problems	4 (12.90)	4 (10.81)	4 (10.81)		12 (11.43)
Extreme problems	4 (12.90)	0 (0.0)	2 (5.41)		6 (5.71)
**Pain/discomfort**					
No problems	11 (35.48)	5 (13.51)	12 (32.43)	0.4253	28 (26.67)
Slight problems	9 (29.03)	12 (32.43)	11 (29.73)		32 (30.48)
Moderate problems	7 (22.58)	13 (35.14)	8 (21.62)		28 (26.67)
Severe problems	4 (12.90)	4 (10.81)	4 (10.81)		12 (11.43)
Extreme problems	0 (0.0)	3 (8.11)	2 (5.41)		5 (4.76)
**Depression/anxiety**					
No problems	19 (61.57)	25 (67.57)	23 (62.16)	0.2675	67 (63.81)
Slight problems	3 (9.68)	8 (21.62)	8 (21.62)		19 (18.10)
Moderate problems	5 (16.13)	1 (2.70)	3 (8.11)		9 (8.57)
Severe problems	1 (3.23)	3 (8.11)	2 (5.41)		6 (5.71)
Extreme problems	3 (9.68)	0 (0.0)	1 (2.70)		4 (3.81)

Bold values are indicate the *p*-values <0.05.

**TABLE 3:** Multiple linear regression for the association between the use of tocilizumab (TCZ) and EQ-5D VAS score.

Variable	β-Estimate	*p-*value	95% Confidence interval	
			Lower	Upper
TCZilizumab	4.26	0.5201	−8.83	17.36
Age	−0.06	0.7937	−0.52	0.40
Gender	7.84	0.5008	−15.20	30.89
Treatment Duration	−0.96	0.4625	−3.54	1.62
Disease Duration	−0.04	0.9290	−0.95	0.87
Number of Comorbidities	0.83	0.7486	−4.29	5.95
Use of Corticosteroids	−9.81	0.3282	−29.64	10.00

Abbreviations: ABA, abatacept; ADA, adalimumab; CRP, baseline C-reactive protein; DAS28, disease activity score; DMARDs, disease-modifying anti-rheumatic drugs; EMRs, electronic health records; ESR, erythrocyte sedimentation rate; ETA, etanercept; HAQ-DI, health assessment questionnaire disability index; HLA, human leukocyte antigen; HRQoL, health-related quality of life; ICERs, incremental cost-effectiveness ratios; mAbs, monoclonal antibodies; MTX, methotrexate; NSAIDs, non-steroidal anti-inflammatory drugs; PROs, patient-reported outcomes; QOL, quality of life; RA, rheumatoid arthritis; RTX, rituximab; TCZ, tocilizumab; VAS, visual analog scale.

## Discussion

This paper pioneers into new ground, examining the impact of TCZ on HRQoL within the specific context of Saudi Arabia, as opposed to contrasting TCZ with commonly utilized biological treatments. Our research presents a compelling insight, patients with RA that received TCZ demonstrated improved HRQoL, gauged through the EQ-5D VAS. However, the consideration of potential covariates diminished the significance of this disparity to non-statistical levels.

Although the characteristics of the patients in this study differ from other previously published real-world studies that compared TCZ to anti-TNF, the findings were not dissimilar (Harrold et al., 2018; Tachkov et al., 2021). In a real-world comparative effectiveness study that compared TCZ monotherapy to anti-TNF biologics plus methotrexate among using RA registry data in the United States, patients with prior exposure to anti-TNF biologics who were started on TCZ monotherapy did not have a better clinical response as measured by the CDAI at 6 months compared to their counterparts on anti-TNF biologics plus methotrexate regardless of methotrexate dose. In addition, a previous investigation conducted in Bulgaria (2012–2016) showed that all examined biological DMARDs resulted in improvement in patient HRQoL using the EQ5D. However, only rituximab appeared to be cost effective in the Bulgarian context (Boyadzieva et al., 2018). Moreover, in another real-world observational study in Bulgaria, the use of biologics appeared to improve the HRQoL after 1-year of treatment with no significant difference between them (Tachkov et al., 2021).

Despite the abundance of studies affirming TCZ efficacy in enhancing physical functionality and HRQoL for RA patients, as previously stated, most of these investigations have predominantly contrasted TCZ with placebo or traditional DMARDs such as methotrexate (Townes et al., 2012). They rarely compared tocilizumab with other biologic therapies such as ADA and ETA among biologic naïve patients (Harrold et al., 2018; Tachkov et al., 2021). However, it is noteworthy that in instances where RA patients responded inadequately to TNF inhibitors like ADA and ETA, TCZ treatment has resulted in immediate and lasting improvements in HRQoL. Greater proportions of PRO improvements were achieved, along with superior disease activity outcomes (Strand et al., 2012; Strand et al., 2018).

The current priority is to conduct a thorough analysis of the cost-effectiveness of using TCZ as a primary therapeutic strategy for managing RA. This is particularly crucial in real-world scenarios, considering its higher acquisition cost. Although our research is, to our knowledge, the first to compare HRQoL among RA patients undergoing TCZ as a primary treatment regimen, it is imperative to consider its limitations. These encompass a relatively small sample size, potential interviewer bias, and possible information bias due to dependency on electronic medical records for patients’ baseline characteristics, and the lack of information about the occupation of the patients which may affect patient HRQoL. Moreover, the patients in the study were on biological DMARDs (TCZ, ADA, and ETA) monotherapy without methotrexate or other conventional synthetic DMARDs, which runs counter to the recommendations of the Saudi Society of Rheumatology. In addition, this is a single-center study which limits the generalizability of the results. Therefore, the findings may not represent the practice in other healthcare institutions (Omair et al., 2022). In addition, the lack of control for disease severity using recognized measurements like the DAS-28 is a substantial limitation.

In summary, the findings of this study indicated no significant differences in QOL among RA patients treated with TCZ *versus* other anti-TNF mAbs, such as ADA and ETA, as evaluated by the EQ-5D-5L questionnaire and VAS score. While patients on TCZ had numerically higher VAS scores, this difference was not statistically significant after adjusting for covariates like age, gender, disease and treatment durations, number of comorbidities, and the use of steroids. In Saudi Arabia, the monthly acquisition cost for the branded subcutaneous formulation of TCZ is $885 based on prices for public healthcare institutions. While the monthly acquisition cost of 40 mg of subcutaneous adalimumab every other week can be as low as $227.87, and $469 for 50 mg subcutaneous etanercept every week for public healthcare institutions. Therefore, the findings of this study may inform discussions about the most cost-effective treatment protocols for RA in Saudi Arabia since the prescription medications costs are fully covered by the public healthcare system. For future endeavors, it is critical to carry out additional studies with enhanced design, a larger sample size, and a more rigorous analysis method. This will secure a more precise comprehension of this phenomenon, hence promoting efficiency in resource allocation for managing RA within the Saudi Arabian healthcare landscape.

## Conclusion

The results of this study contribute to a growing body of research assessing the comparative effectiveness and cost-effectiveness of different biologic treatments for RA. Our findings also underline the need for further studies with larger sample sizes and more robust designs to verify these outcomes and provide a more comprehensive understanding of the impact of various biologics on RA patients’ quality of life. Such studies are crucial in guiding treatment decisions and resource allocation in healthcare settings, particularly in areas where these drugs impose a significant cost burden, like in Saudi Arabia.

## Data Availability

The original contributions presented in the study are included in the article/Supplementary material, further inquiries can be directed to the corresponding author.

## Abbreviations

RA, rheumatoid arthritis; QoL, quality of life; HRQoL, Health-related quality of life; EuroQol, EQ-VAS, Visual analogue scale; mAbs, monoclonal antibodies; HLA, human leukocyte antigen; DAS28, disease activity score; DMARDs, disease-modifying anti-rheumatic drugs; ADA, adalimumab; ETA, etanercept; TCZ, tocilizumab; SC, subcutaneous; ABA, abatacept; RTX, rituximab; EMRs, electronic health records; ESR, erythrocyte sedimentation rate; CRP, C-reactive protein.
